# Beware of Reversal of an Anticoagulated Patient with Factor IX in the Emergency Department: Case Report of a Medical-Legal Misadventure

**DOI:** 10.5811/cpcem.2019.12.43675

**Published:** 2020-01-24

**Authors:** Steven Gannon, David Bell, Kenneth Jackmiczyk, Gregory Moore

**Affiliations:** * Baylor University Medical Center, Pulmonary and Critical Care Medicine, Dallas, Texas; † Maricopa Medical Center, Department of Emergency Medicine, Phoenix, Arizona; ‡ University of Arizona College of Medicine-Phoenix, Department of Emergency Medicine, Phoenix, Arizona; § Mayo Clinic, Department of Emergency Medicine, Rochester, Minnesota

## Abstract

In this article we present a case of a patient who received reversal of anticoagulation therapy with factor IX in violation of hospital guidelines. As a direct result, myocardial infarction and ischemic stroke occurred, leaving the patient neurologically debilitated. Factor IX is indicated in the setting of warfarin-induced, life-threatening bleeding. The patient’s care was provided by an intern with attending physician supervision. Delayed charting and questionable shared decision-making were present in the care. We discuss usage of factor IX, liability for supervision of physicians in training, and factors that can lead to plaintiff awards.

## CASE PRESENTATION

A 54-year-old woman presented to her primary care physician complaining of epistaxis, hematochezia, headache, and a seizure. She had a prior history of seizures. The patient was on warfarin for an unknown reason. Her physician ordered an international normalized ratio (INR), which returned with a result of 13.4 after the patient had gone home. She had been told to hold her warfarin during her office visit. The patient was directed to go immediately to the emergency department (ED) but did not present until the next day. She had a history of very labile INRs in the past, with and without compliance. Multiple prior ED visits with extremely high levels in the past had been treated successfully with vitamin K and fresh frozen plasma (FFP) without complications.

On presentation to the ED the patient complained of a headache. She had no epistaxis or evidence of nasal bleeding. Her skin exam was normal. A neurologic exam was normal. A rectal exam was heme negative and the stool was normal color. The patient had right lower abdominal pain and tenderness. The emergency physician (EP) attending who was board certified in emergency medicine (EM) (seven years experience) supervised an intern (who had just begun residency training) in rendering patient care. A computed tomography (CT) of the brain was done with normal results. An INR lab test returned with a result of greater than 10. Due to abdominal tenderness, the possibility of appendicitis was entertained. A CT of the abdomen was ordered and the surgical service consulted.

The attending EP then discussed the case with the intern and a decision was made to give Profilnine (factor IX). There was no documentation of medical decision-making or discussion with the patient. The decision to administer factor IX was made prior to completion of the surgical consultation or CT result. Subsequently the EP attending left for home before the patient’s consultation or care was completed. He had never administered the drug before and instructed the intern to look up the dose on the Internet and order it. A hospital guideline specifically discussed indications for use of the drug: The patient must have either (1) a serious or life-threatening bleed; or (2) require emergency surgery.

Three hours after administration of the medication the patient developed signs and symptoms of an acute myocardial infarction (MI). An electrocardiogram showed marked ST elevations, which resolved after the administration of tPA. Troponin was elevated as well. Cardiac catheterization performed after resolution of the ST-segment elevation revealed no thrombosis. The patient suffered a cardiac arrest and was subsequently resuscitated. Experts opined that a stroke had also occurred.^1^ She was left in a minimally conscious state with a seven-year life expectancy. A jury rendered a plaintiff verdict for $15 million.

## DISCUSSION

### Dr. Gannon: Caveats when Using Profilnine

Profilnine is the brand name for factor IX complex composed of factors II, IX, X. It has notably low or even nontherapeutic levels of factor VII and thus should not be confused with prothrombin complex concentrate. The primary indications for use are in patients diagnosed with a factor IX deficiency, also known as hemophilia B or Christmas disease. It is indicated in these patients when they present with acute hemorrhage, prophylaxis for bleeding, or in preparation for planned surgical or dental procedures.^2^ Dosing is based both on weight and goal of factor IX level, which in turn is dependent on the severity and/or risk of further bleeding. The cost of factor IX complex is per unit and current available pricing is $1.57 per unit. In a typical 70 kilogram (kg) patient receiving the 75–90 units/kg recommended for treatment of major bleeding, the price for factor IX complex would amount to $8,242.50–$9,891.00.^3^

Known adverse effects from factor IX complex include antibody formation to factor IX, hypersensitivity reactions, thrombotic events, and disseminated intravascular coagulation. While there are no contraindications listed in the manufacturer’s labeling, caution is advised when using factor IX complex in patients with liver disease, history of coronary artery disease, and disseminated intravascular coagulation due to the risk of thromboembolic complications. Factor IX complex has also been used in the treatment of life-threatening hemorrhage associated with warfarin. It is important to note that this use is off-label, and evidence regarding its use for reversal of supratherapeutic INR is poor and heavily expert-opinion based.^4^

Current consensus guidelines do not recommend use of prothrombin complex concentrates outside of the setting of warfarin-associated major bleeding. When it is used for this purpose, the concomitant use of FFP or factor VIIa can be considered as factor IX complex contains nontherapeutic levels of factor VII.^5^ It has been shown, in a small study, to be effective in the treatment of warfarin-associated intracranial hemorrhage without a significantly increased risk of thromboembolic complications when compared to FFP. However, this study did note that reversal with 3-factor prothrombin complex concentrate was accompanied by thrombotic complications (venous thromboembolism, ischemic stroke or MI) in 12.5% of patients.^6^

### Dr. Bell: Medical-Legal Liability of Residents

Between 2009–2013, EM residents were named in 13.4% of malpractice lawsuits.^7^ In a malpractice lawsuit, four elements must be present: duty; breach of duty; causation; and damages. This standard holds true for both residents and attending physicians who are named in the suit. Malpractice cases with residents named were statistically more likely to involve cardiac cases and procedures.^7^

The vexing issue regarding residents is this: What standard of care ought they be held to? Historically, from the 1950s–1980s, residents were held to a lower standard of care than attendings.^8^ However, this has changed over the years in subsequent court rulings. While there is some court variation, in general, medical interns, even though they are unlicensed, are held to the standard of care of a general practitioner who is practicing in a similar setting.^8^ Residents beyond their intern year who are training in a specialty have consistently been found to be held to the higher standard of care of an attending physicians in that specialty.^9^,^10^ In part, this is argued because such residents are licensed and are also presenting themselves to the patients as specialists in a particular field. While there is some possibility that as specialist training progresses, residents are held to increasing stricter standards, there is some variation in the courts regarding this.

Although it can occur that EM residents are the sole practitioners named in malpractice lawsuits, practically speaking this is rare, occurring in only 5.3% of cases between 2009–2013.^11^ Hospitals, training institutions, and attendings are almost always named in malpractice suits as well.^11^ With some minor variations, the courts have consistently found that attending physicians are liable for the residents they are supervising, whether that supervision is in person or at a distance (direct or indirect).^12^

As a malpractice lawsuit progresses it sometimes occurs that those named in the suit with greater financial assets (“deeper pockets”), such as hospitals and attending physicians, are pursued for damages, while residents who may have smaller limits on their malpractice insurance are dropped. However, residency programs should be encouraged and expected to provide appropriate levels of location- and specialty-specific malpractice coverage for their trainees. While it is possible for a resident to argue that they were poorly trained by their program and thus not liable for their malpractice errors, in general such arguments have not succeeded.^9^

### Dr Jackimczyk: Attending Physician Medical-Legal Liability for Residents

Two classic legal cases give insight into liability when attending physicians supervise residents. In Landry v Leonard, a 22-year-old woman was referred by her obstetrician to a teaching hospital for induction of labor. Upon her arrival a fetal stress test was performed by the obstetrical (OB) resident on duty and was interpreted as being normal. The test actually demonstrated fetal distress. The baby was subsequently born neurologically impaired. The supervising attending OB physician was not present at the hospital during the resident’s care of the patient but was named in the lawsuit. It was argued that his lack of appropriate supervision resulted in the child’s brain damage. Both at trial and on appeal the attending obstetrician was dismissed because he never came into contact with the patient. Ultimately, the case went to the Ohio Supreme Court.

The patient had signed consent at the teaching hospital allowing students to administer treatment but the consent also stated that there would be an attending physician delivering general instructions. The Ohio Supreme Court ruled that it was the attending physician’s duty to be present for the birth rather than waiting for the resident to call for help. The Court concluded that a physician-patient relationship arises whenever a physician consents to act for a patient’s benefit. In teaching hospitals a physician-patient relationship may be present when a physician agrees to provide supervision in the care of a patient even if they have no direct or indirect contact with the patient.^13^

In Lownsbury v VanBuren, a 23-year-old woman presented to the ED with a severe headache. She was examined by a resident who ordered a CT of the head. He interpreted the CT as normal and performed a lumbar puncture, which was normal. The patient was admitted to the hospital and was discharged the next day with a diagnosis of muscle tension headache. She was never seen and her case was never reviewed by the attending physician. She returned to the ED the following day with a worsening headache and vomiting. She was examined by an attending physician who noted decreased vision in her left eye. He reviewed the previous day’s CT and noted several small infarcts that the resident hadn’t seen. He repeated the head CT and it showed a “massive cerebral infarct.” She was admitted to the intensive care unit and died three days later.

A lawsuit was filed and the plaintiffs received damages of $500,000, the highest award possible under Louisiana’s malpractice law. The plaintiff’s expert witness noted that the hospital’s bylaws “bar the medical staff from delegating diagnosis and care of patients to practitioners who are not qualified to undertake responsibility and who are not adequately supervised.” The plaintiff’s verdict was upheld on appeal. The attending physician claimed that his lack of involvement in the case eliminated his liability. The court decided that the attending physician, as supervisor of the resident, had accepted a duty to care for the patient and should be held responsible for the error in the resident’s judgment. The court stated that this is the very reason the attending should be present.^14^

### Dr. Moore MD JD: Physician Medical-Legal Mistakes

Plaintiff attorneys, in the setting of possible malpractice, feel very confident in successful litigation when hospital guidelines are violated. Guidelines represent a consensus opinion and result from a variety of authorities agreeing on standard practice. While a physician may disagree with the guidelines, and deviate from them in certain situations, there should be clear documentation of the reasoning behind the deviation. Conversely, when a guideline is followed, plaintiffs admit they have great difficulty proving negligence.

The chart in this case was generated three days later by the attending and seven days later by the intern. In these cases of delayed documentation, when a poor patient outcome occurs, juries can be very skeptical of the truthfulness of the chart. They recognize the documentation may have self-serving and litigation-avoiding purposes.

Recently, plaintiff attorneys have begun to point to a failure of shared decision-making or lack of informed consent when patient outcomes are poor. This is especially true when significant and risky treatment decisions are undertaken without actual or documented involvement of the patient and/or family. A recent randomized controlled simulation using clinical vignettes explored the magnitude of this issue using no/brief/thorough shared decision-making. Of 804 participants, patients who received brief or thorough shared decision-making were 80% less likely to contact a lawyer and expressed higher trust and acceptance of adverse outcomes when compared to patients who received no decision-making.^15^

Ignorance of hospital guidelines, delayed charting, and lack of shared decision-making all played a significant part in this plaintiff outcome of $15 million.

## CONCLUSION

Administration of factor IX is a significant medical decision, which can lead to severe morbidity or mortality. It is also very expensive. It behooves providers to be certain that it is indicated before prescribing this therapy. When supervising others who are using this medication, providers must be aware of their responsibility and liability for others. Optimal charting and information-sharing with patients will reduce liability. Approved guidelines that are in place should be respected and the chart should reflect specific reasons for deviation.

Take Home Points:

1. Use of factor IX can lead to severe morbidity and mortality as well as liability and thus should be given only if specifically indicated.
2. When supervising physicians in training, it should be realized that although those in training may be held liable, the courts have clearly stated that the supervising physician has greatest responsibility and liability.
3. Deviation from accepted clinical guidelines exposes great liability unless there is clear documentation for the thought process and justification that led to the deviation.
4. Delayed charting, when there are bad clinical outcomes, leads to questions of physician honesty by juries when trial occurs.
5. Shared decision-making with patients, when there are significant clinical decisions to be made, reduces liability in resultant negative patient outcomes
